# Correction: Multiplexed genetic engineering of human hematopoietic stem and progenitor cells using CRISPR/Cas9 and AAV6

**DOI:** 10.7554/eLife.43690

**Published:** 2018-11-16

**Authors:** Rasmus O Bak, Daniel P Dever, Andreas Reinisch, David Cruz Hernandez, Ravindra Majeti, Matthew H Porteus

Bak RO, Dever DP, Reinisch A, Hernandez DC, Majeti R, Porteus MH. 2017. Multiplexed genetic engineering of human hematopoietic stem and progenitor cells using CRISPR/Cas9 and AAV6. *eLife* **6**:e27873. doi: 10.7554/eLife.27873.

Published 28, September 2017

In the Material and Methods section of our originally published manuscript, we erroneously defined the concentration of the small molecule StemRegenin 1, (SR1) used for hematopoietic stem and progenitor cell culture, as 0.75mM. The correct concentration for SR1 should be 0.75µM instead of 0.75mM.

The article has been corrected accordingly.

