# Supplementary material for: Multiplexed genetic engineering of human hematopoietic stem and progenitor cells using CRISPR/Cas9 and AAV6
Source: eLife. 2017 Sep 28;6:e27873. doi: 10.7554/eLife.27873 (PMC5656432; doi:10.7554/eLife.27873)
Supplement: Supplementary file 1. — This table summarizes all independent experiments targeting HBB, CCR5, IL2RG, RUNX1, ASXL1, STAG2, and AAVS1 in HSPCs and the reporter genes used. GFP: green fluorescent protein, tNGFR: truncated Nerve Growth Factor Receptor, BFP: blue fluorescent protein. Efficiencies were averaged across 47 independent experiments, N = 47. (b) Overview of genotypes for the non-integrated alleles in mono-genic integration experiments. The three tables show the different INDELs that were identified by Sanger Sequencing of the non-edited allele in mono-genic targeting experiments (CCR5, IL2RG, and RUNX1) used to analyze genotype frequencies shown in Figure 1—figure supplement 2b and d. Alleles are grouped into WT (blue), INDELs that preserve the reading frame (red) and INDELs that disrupt the reading frame (green). Note that INDELs that preserve the reading frame can potentially be disruptive depending on the size and location. For example, the 147 bp deletion in RUNX1 is considered disruptive because of its large size and because it deletes the splice donor site in the intron between exon 2 and 3. For IL2RG, one clone was found to have an allele with integration of 230 bp from the donor (at the end of the RHA and 72 bp into the ITR). (c) Overview of di-genic and biallelic targeting experiments in cord blood (CB), bone marrow (BM), and mobilized peripheral blood (mPB)-derived human CD34+HSPCs. This table summarizes the experiments targeting HSPCs for biallelic and di-genic HR and the reporter genes used. GFP: green fluorescent protein, tNGFR: truncated Nerve Growth Factor Receptor, BFP: blue fluorescent protein. Efficiencies were averaged across 16 and 17 independent experiments, respectively, N = 16 and N = 17. (d) Overview of genotypes for the non-integrated alleles in clones with tri-genic integrations. Each row of the table represents the genotype of a colony established from a tri-genic targeting experiment (IL2RG, HBB, and CCR5). Alleles are grouped into WT (blue), INDELs that [file elife-27873-supp1.pptx]

## Slide 1
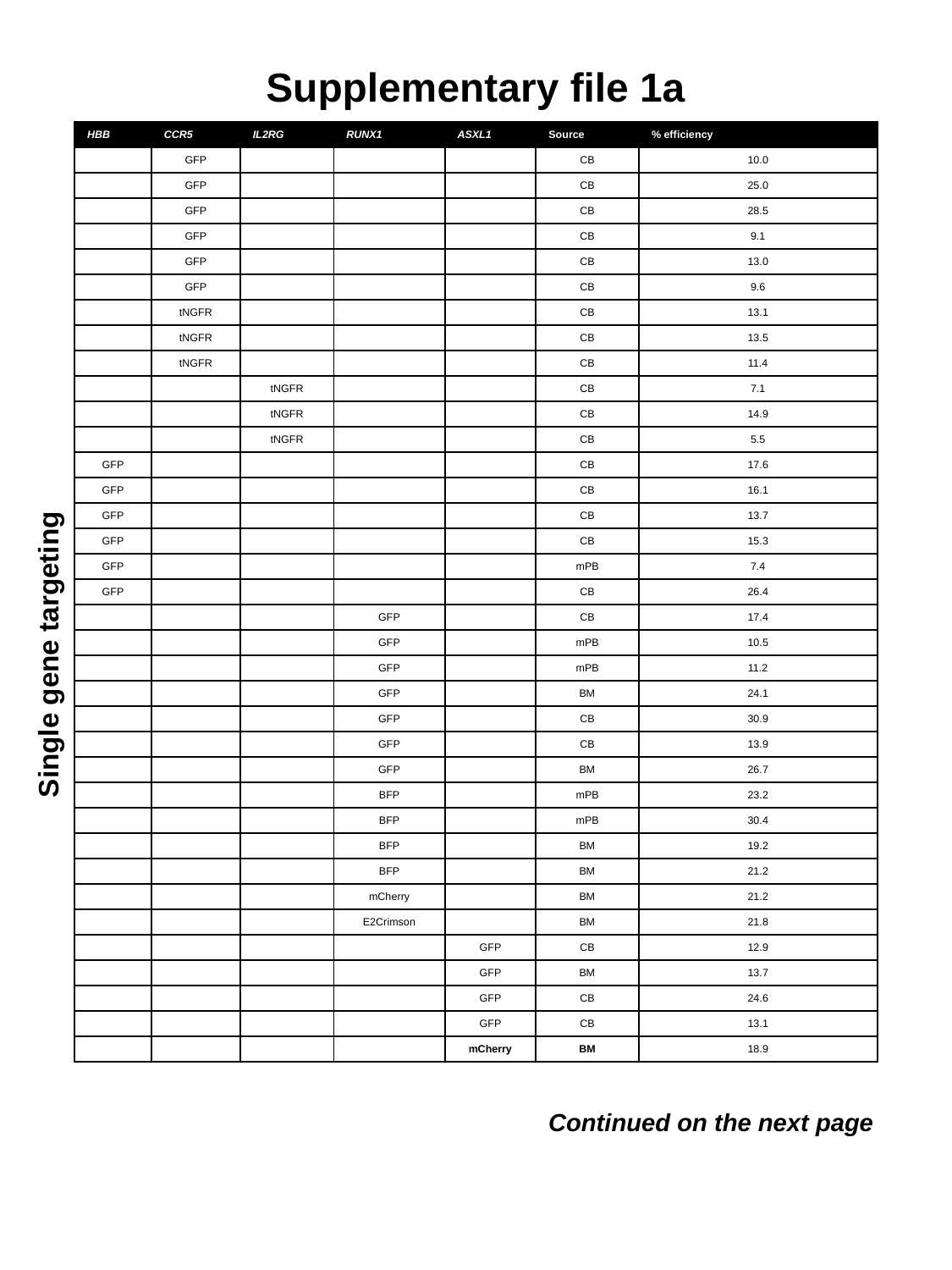

Supplementary file 1a
| HBB | CCR5 | IL2RG | RUNX1 | ASXL1 | Source | % efficiency |
| --- | --- | --- | --- | --- | --- | --- |
| | GFP | | | | CB | 10.0 |
| | GFP | | | | CB | 25.0 |
| | GFP | | | | CB | 28.5 |
| | GFP | | | | CB | 9.1 |
| | GFP | | | | CB | 13.0 |
| | GFP | | | | CB | 9.6 |
| | tNGFR | | | | CB | 13.1 |
| | tNGFR | | | | CB | 13.5 |
| | tNGFR | | | | CB | 11.4 |
| | | tNGFR | | | CB | 7.1 |
| | | tNGFR | | | CB | 14.9 |
| | | tNGFR | | | CB | 5.5 |
| GFP | | | | | CB | 17.6 |
| GFP | | | | | CB | 16.1 |
| GFP | | | | | CB | 13.7 |
| GFP | | | | | CB | 15.3 |
| GFP | | | | | mPB | 7.4 |
| GFP | | | | | CB | 26.4 |
| | | | GFP | | CB | 17.4 |
| | | | GFP | | mPB | 10.5 |
| | | | GFP | | mPB | 11.2 |
| | | | GFP | | BM | 24.1 |
| | | | GFP | | CB | 30.9 |
| | | | GFP | | CB | 13.9 |
| | | | GFP | | BM | 26.7 |
| | | | BFP | | mPB | 23.2 |
| | | | BFP | | mPB | 30.4 |
| | | | BFP | | BM | 19.2 |
| | | | BFP | | BM | 21.2 |
| | | | mCherry | | BM | 21.2 |
| | | | E2Crimson | | BM | 21.8 |
| | | | | GFP | CB | 12.9 |
| | | | | GFP | BM | 13.7 |
| | | | | GFP | CB | 24.6 |
| | | | | GFP | CB | 13.1 |
| | | | | mCherry | BM | 18.9 |
Single gene targeting
Continued on the next page

## Slide 2
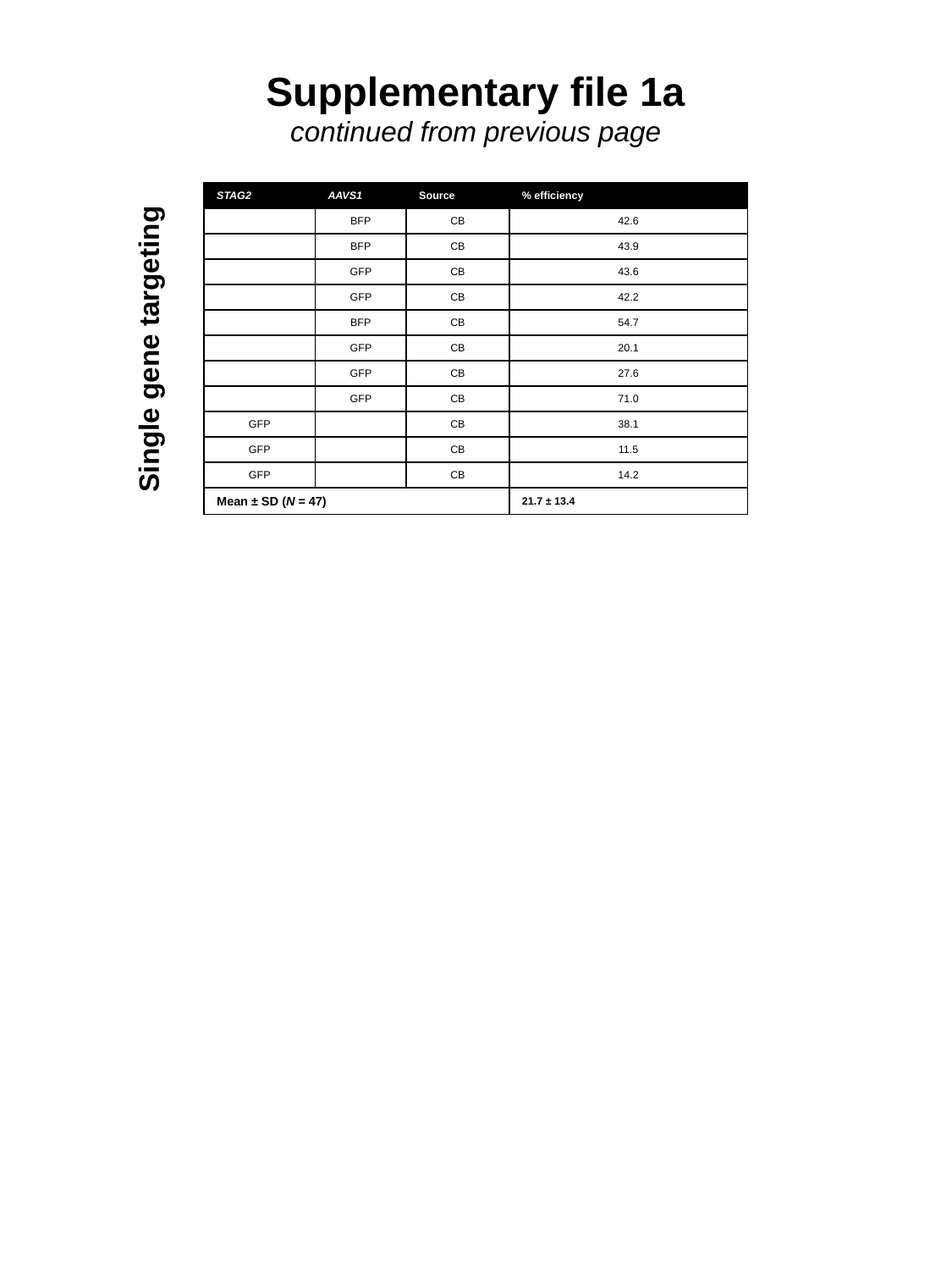

Supplementary file 1a
continued from previous page
| STAG2 | AAVS1 | Source | % efficiency |
| --- | --- | --- | --- |
| | BFP | CB | 42.6 |
| | BFP | CB | 43.9 |
| | GFP | CB | 43.6 |
| | GFP | CB | 42.2 |
| | BFP | CB | 54.7 |
| | GFP | CB | 20.1 |
| | GFP | CB | 27.6 |
| | GFP | CB | 71.0 |
| GFP | | CB | 38.1 |
| GFP | | CB | 11.5 |
| GFP | | CB | 14.2 |
| Mean ± SD (N = 47) | | | 21.7 ± 13.4 |
Single gene targeting

## Slide 3
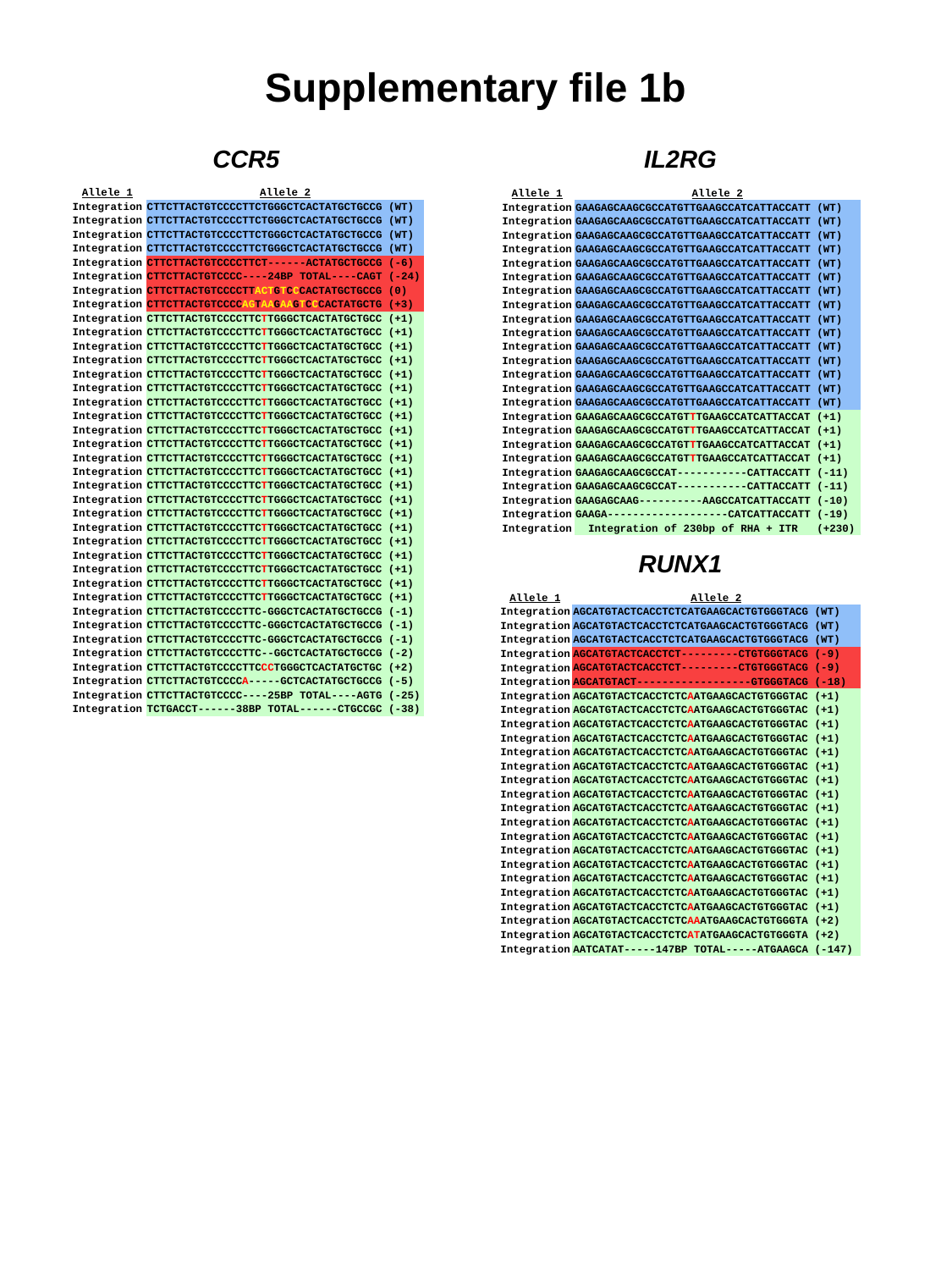

Supplementary file 1b
CCR5
IL2RG
| Allele 1 | Allele 2 |
| --- | --- |
| Integration | CTTCTTACTGTCCCCTTCTGGGCTCACTATGCTGCCG (WT) |
| Integration | CTTCTTACTGTCCCCTTCTGGGCTCACTATGCTGCCG (WT) |
| Integration | CTTCTTACTGTCCCCTTCTGGGCTCACTATGCTGCCG (WT) |
| Integration | CTTCTTACTGTCCCCTTCTGGGCTCACTATGCTGCCG (WT) |
| Integration | CTTCTTACTGTCCCCTTCT------ACTATGCTGCCG (-6) |
| Integration | CTTCTTACTGTCCCC----24BP TOTAL----CAGT (-24) |
| Integration | CTTCTTACTGTCCCCTTACTGTCCCACTATGCTGCCG (0) |
| Integration | CTTCTTACTGTCCCCAGTAAGAAGTCCCACTATGCTG (+3) |
| Integration | CTTCTTACTGTCCCCTTCTTGGGCTCACTATGCTGCC (+1) |
| Integration | CTTCTTACTGTCCCCTTCTTGGGCTCACTATGCTGCC (+1) |
| Integration | CTTCTTACTGTCCCCTTCTTGGGCTCACTATGCTGCC (+1) |
| Integration | CTTCTTACTGTCCCCTTCTTGGGCTCACTATGCTGCC (+1) |
| Integration | CTTCTTACTGTCCCCTTCTTGGGCTCACTATGCTGCC (+1) |
| Integration | CTTCTTACTGTCCCCTTCTTGGGCTCACTATGCTGCC (+1) |
| Integration | CTTCTTACTGTCCCCTTCTTGGGCTCACTATGCTGCC (+1) |
| Integration | CTTCTTACTGTCCCCTTCTTGGGCTCACTATGCTGCC (+1) |
| Integration | CTTCTTACTGTCCCCTTCTTGGGCTCACTATGCTGCC (+1) |
| Integration | CTTCTTACTGTCCCCTTCTTGGGCTCACTATGCTGCC (+1) |
| Integration | CTTCTTACTGTCCCCTTCTTGGGCTCACTATGCTGCC (+1) |
| Integration | CTTCTTACTGTCCCCTTCTTGGGCTCACTATGCTGCC (+1) |
| Integration | CTTCTTACTGTCCCCTTCTTGGGCTCACTATGCTGCC (+1) |
| Integration | CTTCTTACTGTCCCCTTCTTGGGCTCACTATGCTGCC (+1) |
| Integration | CTTCTTACTGTCCCCTTCTTGGGCTCACTATGCTGCC (+1) |
| Integration | CTTCTTACTGTCCCCTTCTTGGGCTCACTATGCTGCC (+1) |
| Integration | CTTCTTACTGTCCCCTTCTTGGGCTCACTATGCTGCC (+1) |
| Integration | CTTCTTACTGTCCCCTTCTTGGGCTCACTATGCTGCC (+1) |
| Integration | CTTCTTACTGTCCCCTTCTTGGGCTCACTATGCTGCC (+1) |
| Integration | CTTCTTACTGTCCCCTTCTTGGGCTCACTATGCTGCC (+1) |
| Integration | CTTCTTACTGTCCCCTTCTTGGGCTCACTATGCTGCC (+1) |
| Integration | CTTCTTACTGTCCCCTTC-GGGCTCACTATGCTGCCG (-1) |
| Integration | CTTCTTACTGTCCCCTTC-GGGCTCACTATGCTGCCG (-1) |
| Integration | CTTCTTACTGTCCCCTTC-GGGCTCACTATGCTGCCG (-1) |
| Integration | CTTCTTACTGTCCCCTTC--GGCTCACTATGCTGCCG (-2) |
| Integration | CTTCTTACTGTCCCCTTCCCTGGGCTCACTATGCTGC (+2) |
| Integration | CTTCTTACTGTCCCCA-----GCTCACTATGCTGCCG (-5) |
| Integration | CTTCTTACTGTCCCC----25BP TOTAL----AGTG (-25) |
| Integration | TCTGACCT------38BP TOTAL------CTGCCGC (-38) |
| Allele 1 | Allele 2 |
| --- | --- |
| Integration | GAAGAGCAAGCGCCATGTTGAAGCCATCATTACCATT (WT) |
| Integration | GAAGAGCAAGCGCCATGTTGAAGCCATCATTACCATT (WT) |
| Integration | GAAGAGCAAGCGCCATGTTGAAGCCATCATTACCATT (WT) |
| Integration | GAAGAGCAAGCGCCATGTTGAAGCCATCATTACCATT (WT) |
| Integration | GAAGAGCAAGCGCCATGTTGAAGCCATCATTACCATT (WT) |
| Integration | GAAGAGCAAGCGCCATGTTGAAGCCATCATTACCATT (WT) |
| Integration | GAAGAGCAAGCGCCATGTTGAAGCCATCATTACCATT (WT) |
| Integration | GAAGAGCAAGCGCCATGTTGAAGCCATCATTACCATT (WT) |
| Integration | GAAGAGCAAGCGCCATGTTGAAGCCATCATTACCATT (WT) |
| Integration | GAAGAGCAAGCGCCATGTTGAAGCCATCATTACCATT (WT) |
| Integration | GAAGAGCAAGCGCCATGTTGAAGCCATCATTACCATT (WT) |
| Integration | GAAGAGCAAGCGCCATGTTGAAGCCATCATTACCATT (WT) |
| Integration | GAAGAGCAAGCGCCATGTTGAAGCCATCATTACCATT (WT) |
| Integration | GAAGAGCAAGCGCCATGTTGAAGCCATCATTACCATT (WT) |
| Integration | GAAGAGCAAGCGCCATGTTGAAGCCATCATTACCATT (WT) |
| Integration | GAAGAGCAAGCGCCATGTTTGAAGCCATCATTACCAT (+1) |
| Integration | GAAGAGCAAGCGCCATGTTTGAAGCCATCATTACCAT (+1) |
| Integration | GAAGAGCAAGCGCCATGTTTGAAGCCATCATTACCAT (+1) |
| Integration | GAAGAGCAAGCGCCATGTTTGAAGCCATCATTACCAT (+1) |
| Integration | GAAGAGCAAGCGCCAT-----------CATTACCATT (-11) |
| Integration | GAAGAGCAAGCGCCAT-----------CATTACCATT (-11) |
| Integration | GAAGAGCAAG----------AAGCCATCATTACCATT (-10) |
| Integration | GAAGA-------------------CATCATTACCATT (-19) |
| Integration | Integration of 230bp of RHA + ITR (+230) |
RUNX1
| Allele 1 | Allele 2 |
| --- | --- |
| Integration | AGCATGTACTCACCTCTCATGAAGCACTGTGGGTACG (WT) |
| Integration | AGCATGTACTCACCTCTCATGAAGCACTGTGGGTACG (WT) |
| Integration | AGCATGTACTCACCTCTCATGAAGCACTGTGGGTACG (WT) |
| Integration | AGCATGTACTCACCTCT---------CTGTGGGTACG (-9) |
| Integration | AGCATGTACTCACCTCT---------CTGTGGGTACG (-9) |
| Integration | AGCATGTACT------------------GTGGGTACG (-18) |
| Integration | AGCATGTACTCACCTCTCAATGAAGCACTGTGGGTAC (+1) |
| Integration | AGCATGTACTCACCTCTCAATGAAGCACTGTGGGTAC (+1) |
| Integration | AGCATGTACTCACCTCTCAATGAAGCACTGTGGGTAC (+1) |
| Integration | AGCATGTACTCACCTCTCAATGAAGCACTGTGGGTAC (+1) |
| Integration | AGCATGTACTCACCTCTCAATGAAGCACTGTGGGTAC (+1) |
| Integration | AGCATGTACTCACCTCTCAATGAAGCACTGTGGGTAC (+1) |
| Integration | AGCATGTACTCACCTCTCAATGAAGCACTGTGGGTAC (+1) |
| Integration | AGCATGTACTCACCTCTCAATGAAGCACTGTGGGTAC (+1) |
| Integration | AGCATGTACTCACCTCTCAATGAAGCACTGTGGGTAC (+1) |
| Integration | AGCATGTACTCACCTCTCAATGAAGCACTGTGGGTAC (+1) |
| Integration | AGCATGTACTCACCTCTCAATGAAGCACTGTGGGTAC (+1) |
| Integration | AGCATGTACTCACCTCTCAATGAAGCACTGTGGGTAC (+1) |
| Integration | AGCATGTACTCACCTCTCAATGAAGCACTGTGGGTAC (+1) |
| Integration | AGCATGTACTCACCTCTCAATGAAGCACTGTGGGTAC (+1) |
| Integration | AGCATGTACTCACCTCTCAATGAAGCACTGTGGGTAC (+1) |
| Integration | AGCATGTACTCACCTCTCAATGAAGCACTGTGGGTAC (+1) |
| Integration | AGCATGTACTCACCTCTCAAATGAAGCACTGTGGGTA (+2) |
| Integration | AGCATGTACTCACCTCTCATATGAAGCACTGTGGGTA (+2) |
| Integration | AATCATAT-----147BP TOTAL-----ATGAAGCA (-147) |

## Slide 4
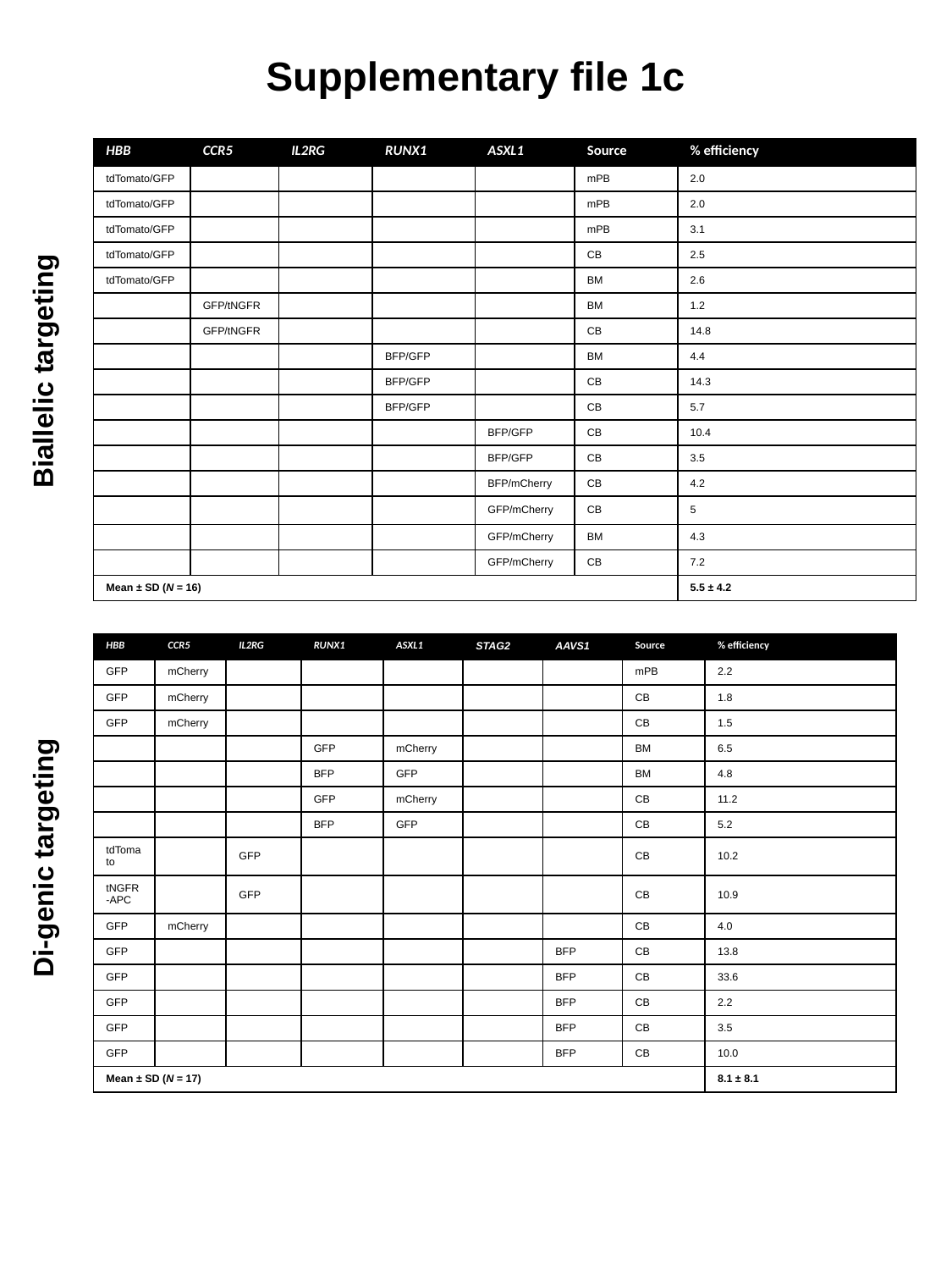

Supplementary file 1c
| HBB | CCR5 | IL2RG | RUNX1 | ASXL1 | Source | % efficiency |
| --- | --- | --- | --- | --- | --- | --- |
| tdTomato/GFP | | | | | mPB | 2.0 |
| tdTomato/GFP | | | | | mPB | 2.0 |
| tdTomato/GFP | | | | | mPB | 3.1 |
| tdTomato/GFP | | | | | CB | 2.5 |
| tdTomato/GFP | | | | | BM | 2.6 |
| | GFP/tNGFR | | | | BM | 1.2 |
| | GFP/tNGFR | | | | CB | 14.8 |
| | | | BFP/GFP | | BM | 4.4 |
| | | | BFP/GFP | | CB | 14.3 |
| | | | BFP/GFP | | CB | 5.7 |
| | | | | BFP/GFP | CB | 10.4 |
| | | | | BFP/GFP | CB | 3.5 |
| | | | | BFP/mCherry | CB | 4.2 |
| | | | | GFP/mCherry | CB | 5 |
| | | | | GFP/mCherry | BM | 4.3 |
| | | | | GFP/mCherry | CB | 7.2 |
| Mean ± SD (N = 16) | | | | | | 5.5 ± 4.2 |
Biallelic targeting
| HBB | CCR5 | IL2RG | RUNX1 | ASXL1 | STAG2 | AAVS1 | Source | % efficiency |
| --- | --- | --- | --- | --- | --- | --- | --- | --- |
| GFP | mCherry | | | | | | mPB | 2.2 |
| GFP | mCherry | | | | | | CB | 1.8 |
| GFP | mCherry | | | | | | CB | 1.5 |
| | | | GFP | mCherry | | | BM | 6.5 |
| | | | BFP | GFP | | | BM | 4.8 |
| | | | GFP | mCherry | | | CB | 11.2 |
| | | | BFP | GFP | | | CB | 5.2 |
| tdTomato | | GFP | | | | | CB | 10.2 |
| tNGFR-APC | | GFP | | | | | CB | 10.9 |
| GFP | mCherry | | | | | | CB | 4.0 |
| GFP | | | | | | BFP | CB | 13.8 |
| GFP | | | | | | BFP | CB | 33.6 |
| GFP | | | | | | BFP | CB | 2.2 |
| GFP | | | | | | BFP | CB | 3.5 |
| GFP | | | | | | BFP | CB | 10.0 |
| Mean ± SD (N = 17) | | | | | | | | 8.1 ± 8.1 |
Di-genic targeting

## Slide 5
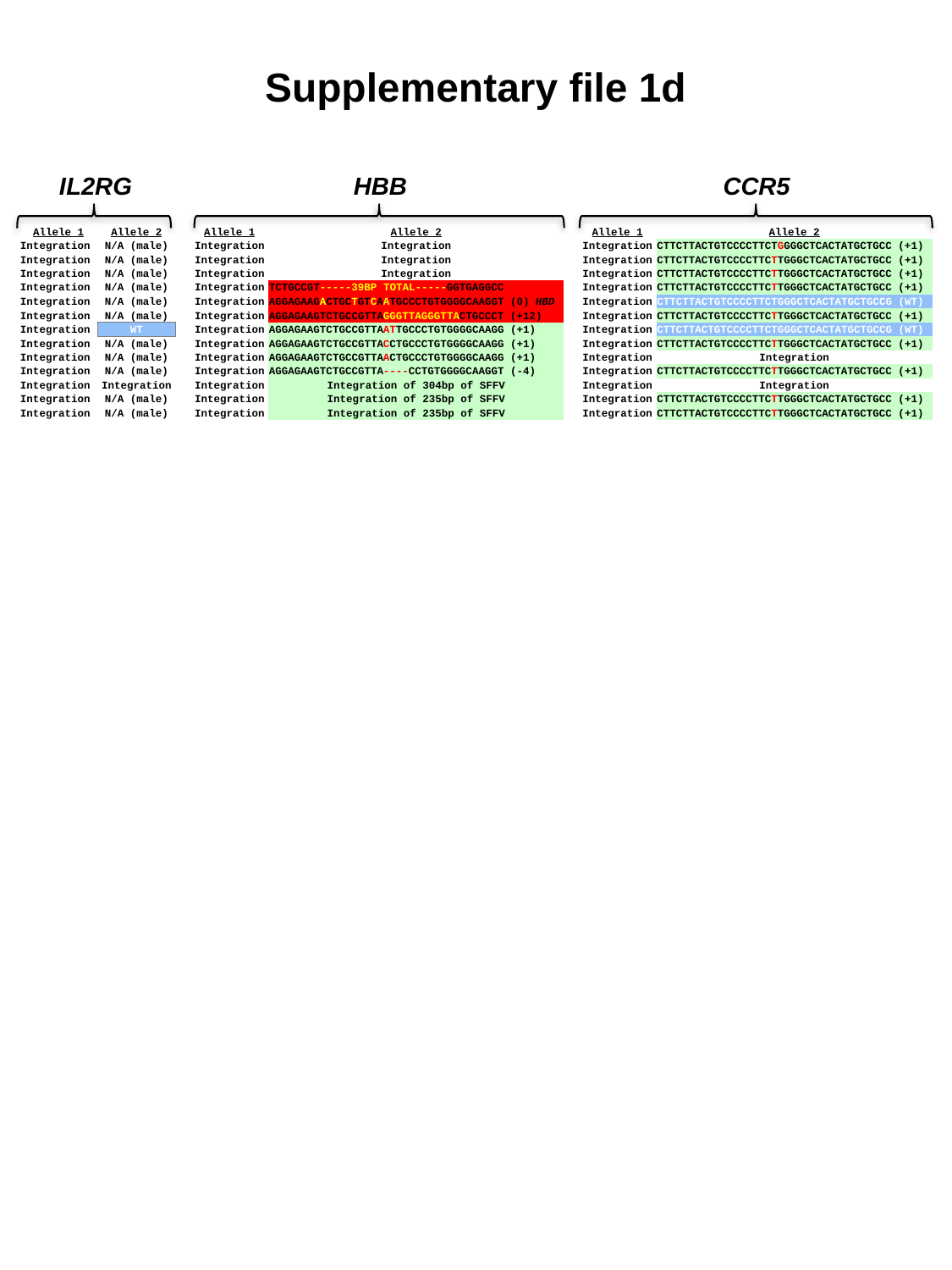

Supplementary file 1d
IL2RG
HBB
CCR5
| Allele 1 | Allele 2 | | Allele 1 | Allele 2 | | Allele 1 | Allele 2 |
| --- | --- | --- | --- | --- | --- | --- | --- |
| Integration | N/A (male) | | Integration | Integration | | Integration | CTTCTTACTGTCCCCTTCTGGGGCTCACTATGCTGCC (+1) |
| Integration | N/A (male) | | Integration | Integration | | Integration | CTTCTTACTGTCCCCTTCTTGGGCTCACTATGCTGCC (+1) |
| Integration | N/A (male) | | Integration | Integration | | Integration | CTTCTTACTGTCCCCTTCTTGGGCTCACTATGCTGCC (+1) |
| Integration | N/A (male) | | Integration | TCTGCCGT-----39BP TOTAL-----GGTGAGGCC | | Integration | CTTCTTACTGTCCCCTTCTTGGGCTCACTATGCTGCC (+1) |
| Integration | N/A (male) | | Integration | AGGAGAAGACTGCTGTCAATGCCCTGTGGGGCAAGGT (0) HBD | | Integration | CTTCTTACTGTCCCCTTCTGGGCTCACTATGCTGCCG (WT) |
| Integration | N/A (male) | | Integration | AGGAGAAGTCTGCCGTTAGGGTTAGGGTTACTGCCCT (+12) | | Integration | CTTCTTACTGTCCCCTTCTTGGGCTCACTATGCTGCC (+1) |
| Integration | WT | | Integration | AGGAGAAGTCTGCCGTTAATTGCCCTGTGGGGCAAGG (+1) | | Integration | CTTCTTACTGTCCCCTTCTGGGCTCACTATGCTGCCG (WT) |
| Integration | N/A (male) | | Integration | AGGAGAAGTCTGCCGTTACCTGCCCTGTGGGGCAAGG (+1) | | Integration | CTTCTTACTGTCCCCTTCTTGGGCTCACTATGCTGCC (+1) |
| Integration | N/A (male) | | Integration | AGGAGAAGTCTGCCGTTAACTGCCCTGTGGGGCAAGG (+1) | | Integration | Integration |
| Integration | N/A (male) | | Integration | AGGAGAAGTCTGCCGTTA----CCTGTGGGGCAAGGT (-4) | | Integration | CTTCTTACTGTCCCCTTCTTGGGCTCACTATGCTGCC (+1) |
| Integration | Integration | | Integration | Integration of 304bp of SFFV | | Integration | Integration |
| Integration | N/A (male) | | Integration | Integration of 235bp of SFFV | | Integration | CTTCTTACTGTCCCCTTCTTGGGCTCACTATGCTGCC (+1) |
| Integration | N/A (male) | | Integration | Integration of 235bp of SFFV | | Integration | CTTCTTACTGTCCCCTTCTTGGGCTCACTATGCTGCC (+1) |

## Slide 6
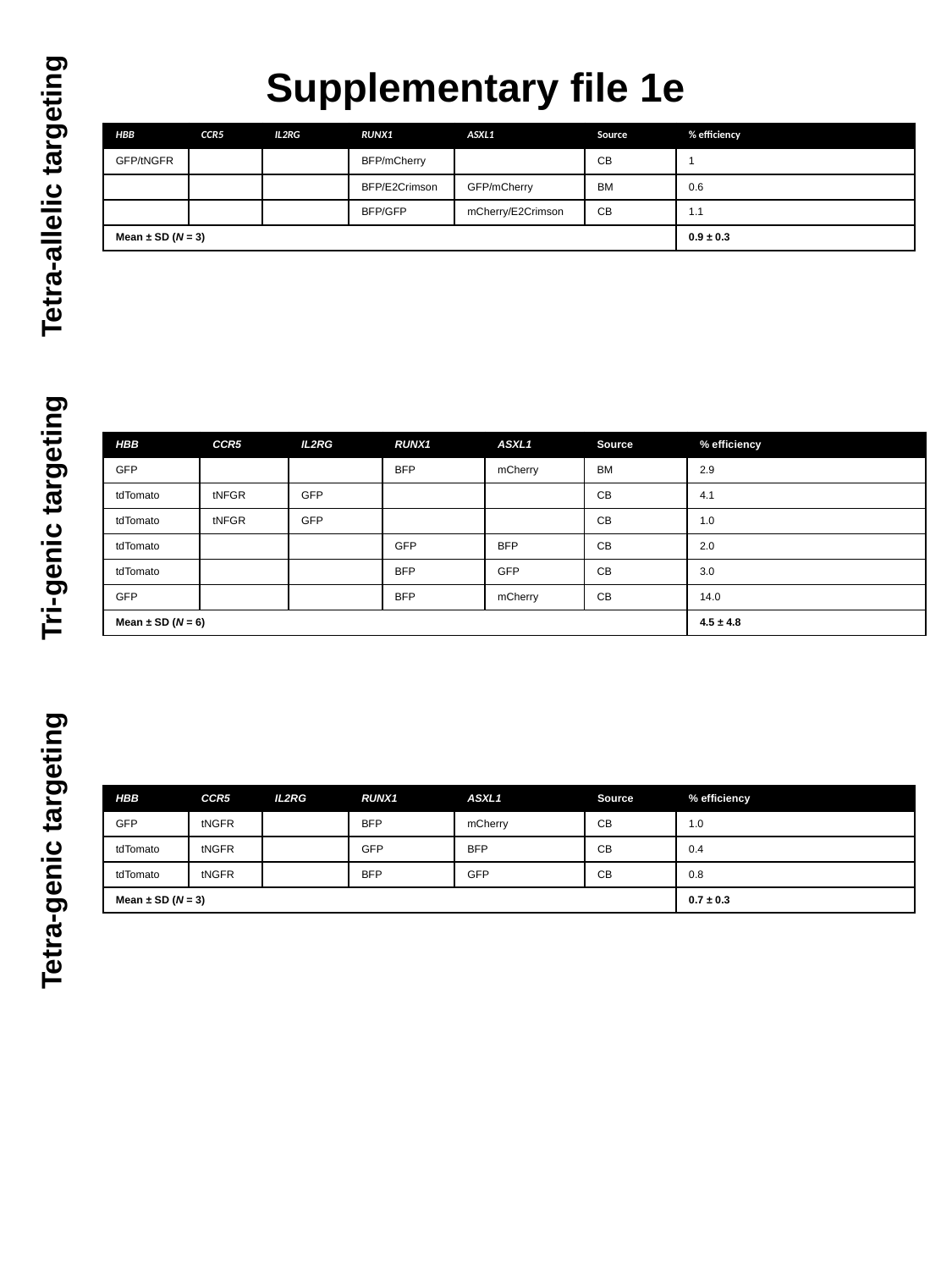

Supplementary file 1e
| HBB | CCR5 | IL2RG | RUNX1 | ASXL1 | Source | % efficiency |
| --- | --- | --- | --- | --- | --- | --- |
| GFP/tNGFR | | | BFP/mCherry | | CB | 1 |
| | | | BFP/E2Crimson | GFP/mCherry | BM | 0.6 |
| | | | BFP/GFP | mCherry/E2Crimson | CB | 1.1 |
| Mean ± SD (N = 3) | | | | | | 0.9 ± 0.3 |
Tetra-allelic targeting
| HBB | CCR5 | IL2RG | RUNX1 | ASXL1 | Source | % efficiency |
| --- | --- | --- | --- | --- | --- | --- |
| GFP | | | BFP | mCherry | BM | 2.9 |
| tdTomato | tNFGR | GFP | | | CB | 4.1 |
| tdTomato | tNFGR | GFP | | | CB | 1.0 |
| tdTomato | | | GFP | BFP | CB | 2.0 |
| tdTomato | | | BFP | GFP | CB | 3.0 |
| GFP | | | BFP | mCherry | CB | 14.0 |
| Mean ± SD (N = 6) | | | | | | 4.5 ± 4.8 |
Tri-genic targeting
| HBB | CCR5 | IL2RG | RUNX1 | ASXL1 | Source | % efficiency |
| --- | --- | --- | --- | --- | --- | --- |
| GFP | tNGFR | | BFP | mCherry | CB | 1.0 |
| tdTomato | tNGFR | | GFP | BFP | CB | 0.4 |
| tdTomato | tNGFR | | BFP | GFP | CB | 0.8 |
| Mean ± SD (N = 3) | | | | | | 0.7 ± 0.3 |
Tetra-genic targeting
